# No findings of SARS-CoV-2 in conjunctival swabs from patients at an emergency outpatient ophthalmological healthcare facility in a Swedish county hospital: a cross-sectional study

**DOI:** 10.1136/bmjophth-2020-000616

**Published:** 2021-03-05

**Authors:** Elisabet Granstam, Anders Krifors, Elisabeth Freyhult, Hanna Åkerblom

**Affiliations:** 1 Center for Clinical Research Region Västmanland, Uppsala University, Västerås, Sweden; 2 Ophthalmology, Region Västmanland, Västerås, Sweden; 3 Physiology and Pharmacology, Karolinska Institute, Stockholm, Sweden; 4 Clinical Microbiology, Region Västmanland, Vasteras, Sweden

**Keywords:** conjunctiva, infection

## Abstract

**Background:**

COVID-19 is caused by SARS-CoV-2. Virus has been found in conjunctiva of hospitalised patients with COVID-19. Conjunctivitis has also been reported as a presenting symptom of disease.

**Objective:**

The aims of the study were to investigate the prevalence of SARS-CoV-2 in the conjunctiva and throat among patients presenting at the emergency outpatient ophthalmological healthcare facility at a county hospital along with investigating the seroprevalence of SARS-CoV-2 among staff at the department.

**Methods and Analysis:**

Swabs from conjunctiva and throat of patients were analysed with real-time reverse transcriptase PCR (RT-PCR) for SARS-CoV-2. Blood samples for serological analysis were obtained from staff. A questionnaire was used to investigate symptoms associated with COVID-19 during the last 3 months as well as symptoms for which the patients were seeking ophthalmological healthcare.

**Results:**

In total, 68 patients and 70 individuals from the staff were included in the study. Conjunctivitis was observed in 7% of patients. One patient, presenting with reduced visual acuity due to preretinal haemorrhage in the macula, was positive for SARS-CoV-2 in throat swab. Contact tracing was negative. All other RT-PCR tests were negative. Seropositivity for SARS-CoV-2 was found in 4% of staff.

**Conclusions:**

Our study demonstrated low prevalence of SARS-CoV-2 among patients as well as low seroprevalence of SARS-CoV-2 IgG-antibodies among staff at the ophthalmological ward. The risk for contracting COVID-19 at the department was small. Follow-up investigation is planned.

**Key messages**

**What is already known about this subject?**

- SARS-CoV-2 virus has been found in conjunctiva of hospitalised patients with COVID-19.

**What are the new findings?**

- Low prevalence of SARS-CoV-2 measured using real-time reverse transcriptase PCR of swabs from conjunctiva and throat in patients presenting at the outpatient emergency facility at a Swedish county hospital as well as low seroprevalence of SARS-CoV-2 IgG-antibodies among staff at the ophthalmological ward was found.

**How might these results change the focus of research or clinical practice?**

- Low exposure and low risk of contracting COVID-19 at the facility were demonstrated. Protection against airborne spreading and close contact with infected persons continue to be highly relevant.

## Introduction

COVID-19 was first reported from Wuhan, Hubei, China, in December 2019. COVID-19 is caused by SARS-CoV-2.^1^ The disease primarily affects the respiratory tract, but manifestations from other organ systems have been reported, such as gastrointestinal and neurological symptoms. In hospitalised patients with moderate-to-severe COVID-19, conjunctivitis has been reported in one-third of patients.^2^ In a case-report, SARS-CoV-2-related follicular conjunctivitis was described.^3^ SARS-CoV-2 has been detected in conjunctival swabs from patients with COVID-19 admitted to hospital,^2 4^ although conflicting results have been reported.^5^

In a questionnaire-based study of 56 previously hospitalised patients with COVID-19, 27% of patients reported ocular symptoms in the course of the disease.^6^ In six out of 15 individuals, ocular symptoms appeared before the onset of fever or respiratory symptoms.^6^ Further, in five patients from Italy, conjunctivitis was the presenting and only symptom of COVID-19.^7^ The prevalence of SARS-CoV-2 among patients presenting at an outpatient ophthalmological clinic is not known.

Receptors for SARS-CoV-2 are present in the conjunctiva representing a potential route of infection for the virus.^8^ Furthermore, contact with conjunctival secretions containing the virus has been suggested to impose a risk for contracting COVID-19. Concerns have been raised that hospital staff at an ophthalmological ward might be at particular risk due to potential exposure during ophthalmological examinations.^9^ Seroprevalence of SARS-CoV-2 IgG-antibodies has been reported from a general hospital staff in Belgium^10^ and Sweden,^11^ but so far there have been no specific reports on seroprevalence among ophthalmic staff.

Therefore, the aim of the present study was to investigate the prevalence of SARS-CoV-2 in the conjunctiva and throat among patients presenting at the emergency outpatient ophthalmological healthcare facility at a county hospital situated in the greater Stockholm area, which has been hit hard by COVID-19. We also aimed to investigate the seroprevalence of SARS-CoV-2 among the staff of the ophthalmological department.

## Materials and methods

### Study subjects

All patients presenting at the emergency outpatient ophthalmological healthcare facility between 4 June and 15 June 2020 were informed about the study and asked to participate. All enrolled patients gave their written consent. Study procedures included swabs from conjunctiva and throat for real-time reverse transcriptase PCR (RT-PCR) for SARS-CoV-2 and a questionnaire investigating symptoms associated with COVID-19 during the last 3 months as well as symptoms for which the patients were seeking ophthalmological healthcare. The questionnaire followed the recommendations from the WHO for COVID-19 studies.^12^

All staff at the department of ophthalmology, Region Västmanland Sweden, were informed about the study and asked to participate. All enrolled individuals gave their written consent. Study procedures included blood samples for serological analysis of antibodies against SARS-CoV-2 and a questionnaire investigating symptoms associated with COVID-19 during the last 3 months. The questionnaires for patients and staff are provided as online supplemental material.

10.1136/bmjophth-2020-000616.supp1 Supplementary data

10.1136/bmjophth-2020-000616.supp2 Supplementary data

### SARS-CoV-2 RT-PCR and serological tests

Real-time RT-PCR tests for SARS-CoV2 nucleic acid were performed on conjunctival and throat swabs using either Allplex SARS-CoV-2 PCR (CFX96 instrument) or Abbott SARS-CoV-2 PCR (m2000 instrument) in accordance with the manufacturers’ instructions.^13 14^ A positive result was confirmed using Cepheid Xpert Xpress SARS-CoV-2.^15^ After collection, the swabs were promptly transported to the local laboratory and stored in a refrigerator at +5°C (temperature limits +2 to +8) until analysed.

Serological testing was conducted using the Abbott SARS-CoV-2 IgG assay (ARCHITECT instrument), a chemiluminescent microparticle immunoassay used for the qualitative detection of antibodies towards the nucleocapsid protein of SARS-CoV-2 (sensitivity 100%, specificity 99.6%).^16 17^ All positive tests were then confirmed by using the Euroimmune Anti-SARS-CoV-2 Assay, an ELISA for the detection of IgG against the SARS-CoV-2 spike protein.

Data are presented with descriptive statistics (IBM SPSS Statistics, V.25.0 IBM Corp).

Patients or the public were not involved in the design, or conduct, or reporting, or dissemination plans of our research.

## Results

### RT-PCR for SARS-CoV-2 in patients

In total, out of 80 adult patients coming to the outpatient emergency facility, 68 patients were included in the study; see table 1.

**Table 1:** Age, gender and main diagnosis of the 68 patients presenting at the emergency outpatient ophthalmological healthcare facility between 4 June and 15 June

Patient demographics	
Age (years)	
Mean (SD), range	58.7 (18.7), 20–95
Gender	
Male/female	36/32
Diagnosis n (%)	
Dry eye/blepharitis	3 (4%)
Conjunctivitis	5 (7%)
Corneal erosion	5 (7%)
Corneal foreign body	8 (11%)
Keratitis	3 (4%)
Episcleritis/iritis	7 (10%)
Posterior vitreous detachment	10 (14%)
Vitreous haemorrhage	3 (4%)
Retinal rupture/detachment	5 (7%)
Trauma	3 (4%)
Other	16 (24%)

The term ‘other’ refers to single cases of varying events, for example, endophthalmitis, vasculitis, exudative macular degeneration, third nerve palsy and branch retinal vein occlusion.

Ten patients declined participation in the study, one patient had already been included at a previous visit and one patient was excluded due to irrigation after a suspected chemical injury following which a conjunctival swab was impossible to obtain.

In total, 71% of the patients reported one or more symptoms from the respiratory tract during the last 3 months. Most frequently reported symptoms were: shortness of breath (58% of patients), nasal congestion (48%), runny nose (48%) and sneezing (38%). All samples from the conjunctiva and throat were successfully analysed. One patient had a positive result in the throat swab but a negative result in the swab from the conjunctiva. All other RT-PCR tests were negative for SARS-CoV-2.

### Case report

**Figure 1:**
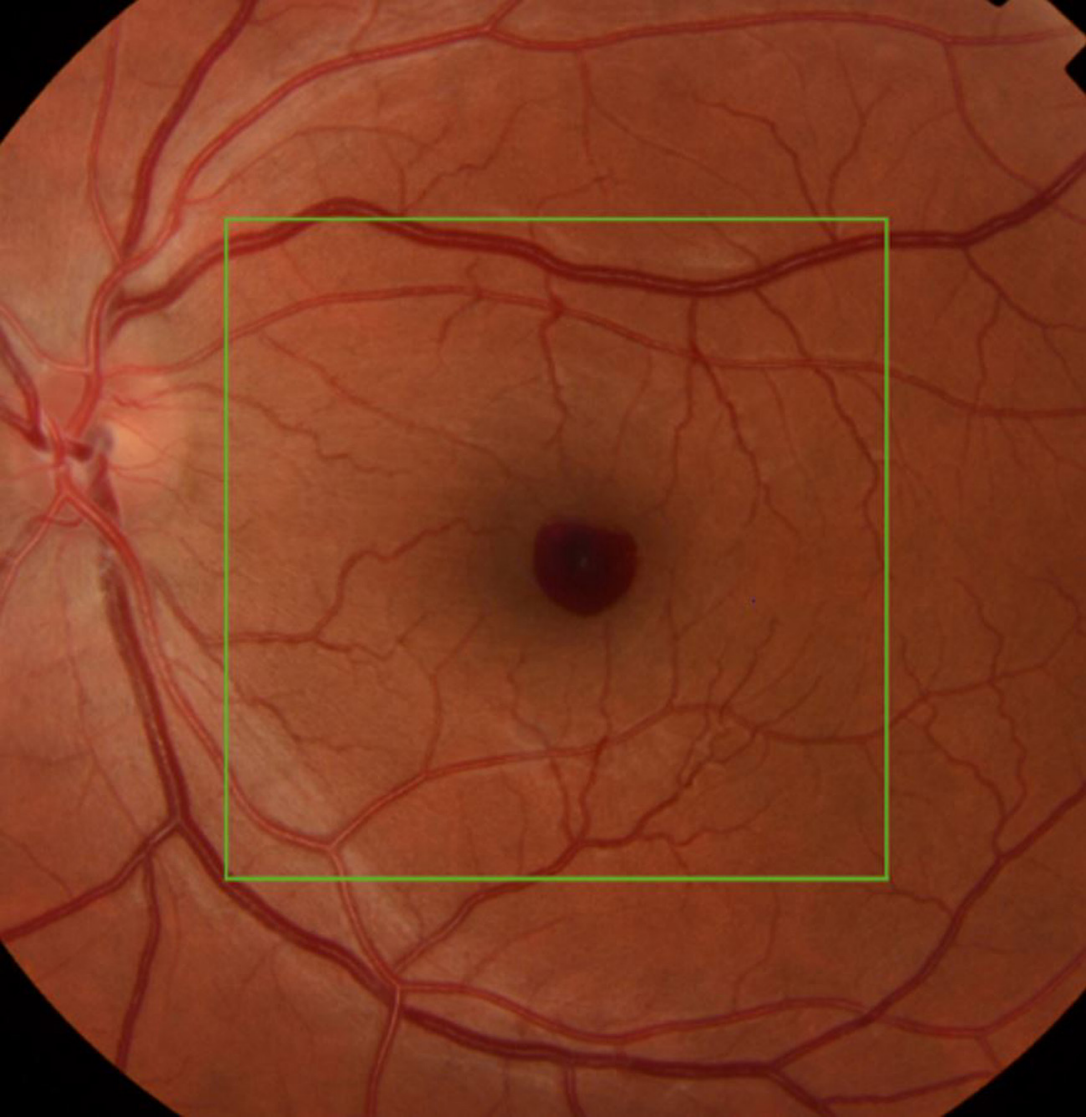
Fundus appearance in the 31-year-old man presenting at the department of ophthalmology due to rapid onset of reduced visual acuity in left eye. A small preretinal haemorrhage in the macula was found.

**Figure 2:**
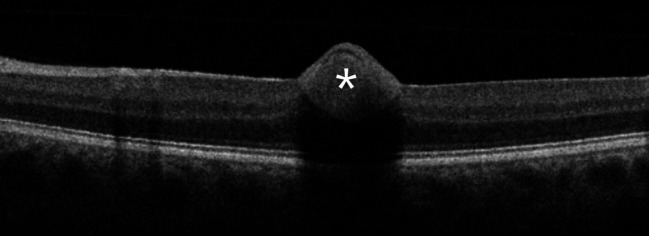
Optical coherence tomography (OCT) with Spectral-domain (SD)-OCT (Topcon OCT 2000) in the macular area in the 31-year-old man presenting at the department of ophthalmology due to rapid onset of reduced visual acuity in left eye. A preretinal haemorrhage in the macula was found. *Preretinal haemorrhage.

A 31-year-old man presented due to rapid onset of reduced visual acuity in the left eye. Visual acuity in the left eye at presentation was 0.2 Snellen visual acuity chart (20/100). Intraocular pressure was normal (13 mm Hg). There were no signs of intraocular inflammation in the eye. Fundus examination revealed a localised preretinal haemorrhage in the macula (figure 1), also seen on optical coherence tomography (figure 2). There were no findings of posterior vitreous detachment, retinal rupture, retinal vasculitis or other pathology in the eye. Findings from the right eye were unremarkable. In the questionnaire, the patient reported having had fever, fatigue, coughing and sneezing, a sore throat, shortness of breath and anosmia during the previous 3 months, but he did not report ongoing fever or symptoms from the respiratory tract. He had not previously had PCR for SARS-CoV-2. The patient was positive for SARS-CoV-2 in the throat swab but not in the conjunctival swab. He was informed about test results and relevant contact tracing was performed in accordance with Swedish law. Contact tracing among staff was negative for SARS-CoV-2.

### Serology in hospital staff

Out of 81 employees at the department of ophthalmology, 70 individuals were included in the study for serological analysis of IgG antibodies against SARS-CoV-2. Eleven nurses, who had been relocated and worked at COVID-19 wards at the hospital, were not included in the study. The occupation and proportion of staff with direct patient contact are presented in table 2.

**Table 2:** Occupation and proportion of direct patient contact among the staff of the department of ophthalmology

Occupation	Number of individuals	Number of positive SARS-CoV-2 IgG
Assistant nurse	10	1
Nurse	21	1
Orthoptist/optician	7	0
Physician	14	1
Administrative staff	18	0
Total number	70	3
Patient contact	52 (74%)	3
No patient contact	18 (26%)	0

Nine persons in the staff had previously had a swab test for COVID-19 and two had been positive for SARS-CoV-2. These two persons and one additional individual had positive results for antibodies against SARS-CoV-2 (4%). All samples were tested with the Abbott SARS-CoV-2 IgG assay, as it has higher sensitivity than the Euroimmune Anti-SARS-CoV-2 Assay.^16 17^ Three samples were positive in the Abbott assay (nucleocapsid target), and they were then confirmed by using the EuroImmune IgG ELISA assay (spike protein target). All other tests were negative.

In total, 90% of the staff reported having had one or more of the symptoms in the survey during the last 3 months, most commonly sneezing (61%), headache (57%), fatigue (56%), nasal congestion (51%), runny nose (48%) and sore throat (41%). More than half off the staff reported having been absent from work due to illness during the same time period but none had required inpatient hospital care.

## Discussion

In this study, we found a low prevalence of SARS-CoV-2 among patients presenting at the emergency outpatient ophthalmological healthcare facility at the county hospital, indicating low exposure of SARS-CoV-2. Additionally, we demonstrated low seroprevalence of SARS-CoV-2 IgG-antibodies among hospital staff at the ophthalmological ward.

The county of Västmanland is situated in the greater Stockholm area and has been hit hard by SARS-CoV-2. The incidence of COVID-19 as per 6 July 2020 was 873 per 100 000 inhabitants in Västmanland and 902 per 100 000 inhabitants in Stockholm, respectively.^18^ Nevertheless, few patients in need for emergency ophthalmological healthcare were infected with SARS-CoV-2, suggesting good adherence to the recommendations by the healthcare authorities of social distancing in all situations and avoidance of any contacts in case of fever or respiratory tract symptoms.^18^

In case patients attending the clinic presented at the reception desk with symptoms suggestive of COVID-19, they were immediately asked to return home and the appointment at the eye clinic was postponed. At the time of the study, precautionary measures included physical distancing during all aspects of the appointment at the clinic, also in the waiting areas. All slit lamps had been supplied with protective plastic shields. Frequent use of alcohol disinfectants on all equipment, with which the patients had been in contact during the appointment, was applied. Facial masks or visors were not in use at this time neither by healthcare professionals nor by patients as according to recommendations by the healthcare authorities.

Conjunctivitis was diagnosed in 7% of patients in our study. None of these patients were positive for SARS-CoV-2 in either the conjunctival or throat swab, suggesting that the cause of conjunctivitis was other than infection with SARS-CoV-2 in our patients although this cannot be completely ruled out. In five Italian patients with lengthy conjunctivitis without improvement for several days, PCR on nasopharyngeal swabs was positive for SARS-CoV-2.^7^ Conjunctivitis remained the only symptom of COVID-19 in these patients and no patient developed fever or respiratory symptoms.^7^ Additionally, in a retrospective review of patients, who had previously been hospitalised with COVID-19, 6 out of 56 individuals (11%) reported ocular symptoms such as sore eyes, itching, foreign body sensation, tearing, redness, dry eyes, eye secretions and floaters up to 7 days before the onset of fever or respiratory symptoms.^6^ Further, in the study from the Hubei province in China in hospitalised patients with at least moderately severe COVID-19, conjunctivitis was reported in almost a third of the patients.^2^ SARS-CoV-2 was detected on RT-PCR in both conjunctival and nasopharyngeal swabs in two out of 12 patients with ocular symptoms, indicating that virus can be discharged in conjunctiva and tears in critically ill patients.^2^ Taken together, the findings suggest that high-level attention should be applied when meeting patients with symptoms of conjunctivitis in the times of the pandemic.

One patient was found to be positive for SARS-CoV-2 in the throat swab. This patient presented with a sudden reduction in visual acuity, which turned out to be caused by a preretinal haemorrhage in the macular area. No risk factors for preretinal haemorrhage were identified in this patient. Retinal vascular events have been reported in conjunction with other systemic viral infections.^19^ Further, in a case series of patients with severe COVID-19, 10 out of 18 patients had retinal abnormalities such as flame-shaped haemorrhages or cotton-wool spots, suggesting involvement of retinal vasculature.^20^ Although our patient was not critically ill, it cannot be ruled out that the haemorrhage was related to the SARS-CoV-2 infection. Contact tracing after the positive PCR-test was obtained did not reveal any transmission to hospital staff.

The seroprevalence of SARS-CoV-2 IgG-antibodies was low, 4%, among hospital staff at the department of ophthalmology. A much higher seroprevalence of 19% among healthcare staff was reported from a hospital in Stockholm^11^ whereas prevalence of SARS-CoV-2 IgG antibodies in hospital staff at a tertiary centre in Belgium was 6.4%.^12^ The findings indicate large variations in exposure and seroconversion among hospital staff also between relatively closely situated hospitals. The low seroprevalence at our department likely mirrors the low prevalence of SARS-CoV-2 infection in our patients and is in line with good adherence to recommendations by the healthcare authorities also by the hospital staff. It is also important to bear in mind the possibility of ‘seroreversion’. Such loss of seropositivity over time was found in 12.2% of initially seropositive participants in a study from Tokyo, Japan, by means of repeated testing over 3–5 weeks.^21^ Our questionnaire showed that a majority of the staff reported symptoms which could be associated with COVID-19 and had subsequently stayed home from work.

The findings indicate that the general precautions taken at the ophthalmological ward including social distancing, plastic shields on the slit lamps, hand disinfection and careful use of alcohol disinfectant on all contact surfaces were sufficient to prevent transmission.

One strength of our study is the cross-sectional design with simultaneous PCR testing of patients and serological testing of the staff at the department. Another strength is a high willingness to participate in the study, and only very few patients and none of the staff declined to participate.

Even though almost all consecutive patients were included during the study period, one weakness was the small sample size, which has to be taken into account when interpreting the results. The absence of a positive RT-PCR test from the conjunctiva and throat in our study participants may be attributed to chance and not represent the true prevalence. A larger sample of patients would have yielded a more accurate estimate of the true prevalence. On the other hand, the patients demonstrated variability of diagnoses and were considered a representative selection of patients at the department. It should also be emphasised that the study population consisted of individuals at the outpatient facility; the patients were allowed to attend the clinic only after screening questions suggestive of COVID-19 were negative, reducing the risk of COVID-19 among participants.

The serological tests used in the study were able to correctly recognise the two individuals from the staff that were previously known to have been COVID-19 PCR positive. The combined use of two different and highly specific serological tests provides a high positive predictive value, even in a low prevalence population such as ours.

No previous asymptomatic COVID-19 infections were identified but cannot be entirely ruled out, as a recent study suggests a rapid loss of antibodies among patients with asymptomatic COVID-19.^22^ Also, the potential role of a T-cell mediated immunologic response to COVID-19 may lead to an underestimation of prior exposure to the virus. However, the vast majority of COVID-19 symptomatic patients develop antibodies^23^ and a potential T-cell response is unlikely to significantly alter the findings of this study.

In conclusion, a low prevalence of SARS-CoV-2 among patients and low prevalence of seropositivity for SARS-CoV-2 among ophthalmological staff were demonstrated, indicating low exposure and low risk for contracting SARS-CoV-2 at the department of ophthalmology at present. Protection against airborne spreading and close contact with infected persons, the main transmission routes, continues to be highly relevant.^24^ Since the situation is rapidly evolving, a repeated measurement in patients and staff is planned to closely follow the development.
