# Supplementary material for: No findings of SARS-CoV-2 in conjunctival swabs from patients at an emergency outpatient ophthalmological healthcare facility in a Swedish county hospital: a cross-sectional study
Source: BMJ Open Ophthalmol. 2021 Mar 5;6(1):e000616. doi: 10.1136/bmjophth-2020-000616 (PMC7938470; doi:10.1136/bmjophth-2020-000616)
Supplement: Supplementary data [file bmjophth-2020-000616supp001.pdf]

Questionnaire – patients

1. General information:

No.	
Gender	☐ Female ☐ Male
Previous test for SARS-CoV-2 RNA PCR	☐ No ☐ Yes ☐ Pos. ☐ Neg
Date of previous test	

2. Symptoms the last three months:

Fever > 38 C	☐ Yes ☐ No
Chills	☐ Yes ☐ No
Fatigue	☐ Yes ☐ No
Muscle aches	☐ Yes ☐ No
Cough	☐ Yes ☐ No
Shortness of breath	☐ Yes ☐ No
Sore throat	☐ Yes ☐ No
Runny nose	☐ Yes ☐ No
Nasal congestion	☐ Yes ☐ No
Difficulty breathing	☐ Yes ☐ No
Sneezing	☐ Yes ☐ No
Chest aches	☐ Yes ☐ No
Other respiratory problem	☐ Yes ☐ No
Headache	☐ Yes ☐ No
Nausea/Vomiting	☐ Yes ☐ No
Stomachache	☐ Yes ☐ No
Diarrhea	☐ Yes ☐ No
Loss of appetite	☐ Yes ☐ No
Hospital care	☐ Yes ☐ No
Absence from work	☐ Yes ☐ No

3. Eye symptoms the last two weeks

Redness	☐ Yes ☐ No
Stickiness	☐ Yes ☐ No
Increased tearing	☐ Yes ☐ No
Sensitivity to light	☐ Yes ☐ No
Irritation	☐ Yes ☐ No
Pain	☐ Yes ☐ No
Decreased vision	☐ Yes ☐ No
Other:	
